# The accuracy and safety of using the electrocardiogram positioning technique in localizing the peripherally inserted central catheter tip position: A systematic review and meta‐analysis

**DOI:** 10.1002/nop2.932

**Published:** 2021-06-16

**Authors:** Chen Yu, Luo Shulan, Wang Juan, Lin ling, Luo Chun‐Mei

**Affiliations:** ^1^ Department of Orthopaedics Xinqiao Hospital The Army Medical University Chongqing China

**Keywords:** electrocardiogram, peripherally inserted central catheter tip, tip position

## Abstract

**Aim:**

We performed a systematic review and meta‐analysis to examine the accuracy and safety of using the electrocardiogram (ECG) positioning technique to localize the peripherally inserted central catheter (PICC) tip position to provide objective evidence for its clinical application.

**Methods:**

We searched the literature for randomized controlled trials evaluating the diagnostic analysis of using electrocardiograms to localize PICC tip positions in the MEDLINE, EMBASE, PubMed, Cochrane Central Register of Controlled Trials (CENTRAL) and PsycINFO databases. We used a risk ratio with accompanying 95% confidence interval (CI) to express estimates. Reviewer Manager (RevMan) 5.1.0 was used to complete all statistical analyses.

**Results:**

This systematic review identified 9 studies (*N* = 3,194 patients). Overall, the results of the meta‐analysis revealed that, for patients in whom the ECG positioning method was used compared with patients in whom the landmark positioning method was used, the RR for accurate catheter tip positioning was RR = 1.17, and the difference was statistically significant. The RR for the incidence of complications was RR = 0.28, and the difference was statistically significant.

**Conclusions:**

The application of ECGs in PICC tip positioning can improve the accuracy of catheter tip positioning and reduce the incidence of related complications.

**Summary Statement**

**1. What is already known about this topic? (include key points and/or knowledge gaps)**.

Reference to a landmark location on the body surface is a commonly used method to predict the position of the catheter. However, in recent years, the electrocardiogram (ECG) localization technique has been used due to its simplicity, ease of learning, dynamic monitoring and real‐time positioning as well as for its ability to reduce financial burden and avoid unnecessary radiation damage. However, which method is better is highly controversial.

**2. What this paper adds: (research findings/key new information)**.

Our research shows that the application of the atrial ECG in PICC tip positioning can improve the accuracy of catheter tip positioning, reduce the incidence of related complications and provide a scientific basis for further promotion of the atrial ECG positioning method. This way is better than landmark location.

**3. The implications of this paper: (how findings influence or can be used to change policy/practice/research/education)**.

ECGs could improve the accuracy of PICC tip localization and reduce the incidence of related complications. It is suitable for infants, pregnant women and elderly patients.

## 1 BACKGROUND

The final placement of the peripherally inserted central catheter (PICC) tip is in the lower third of the superior vena cava or the junction of the superior vena cava and the right atrium (Mary, 2011). The position of the catheter tip is the key factor influencing the effect, duration and complication rate of catheter use, and the most severe adverse effect of a misplaced central venous line is cardiac tamponade, which has a mortality rate of 70% (Hostetter et al., 2010). Therefore, it is imperative that the catheter tip sits in an adequate position within the central circulation to reduce potential complications (Johnston et al., 2013). Reference to a landmark location on the body surface is a commonly used method to predict the position of the catheter. Although the method of body surface positioning using a landmark is simple, convenient and economical, during operation, it is difficult to determine whether the catheter is inserted in a wrong place in a timely manner if it is caused by measurement error: vascular malformation or placement in other non‐target veins. In recent years, the electrocardiogram (ECG) localization technique has been used due to its simplicity, ease of learning, dynamic monitoring and real‐time positioning as well as for its ability to reduce financial burden and avoid unnecessary radiation damage. In clinical practice, there are many studies on the application of ECGs for PICC tip localization, but there are no evidence‐based clinical practice guidelines to suggest its accuracy and superiority (Oliver & Jones, 2014; Rosche & Stehr, 2018).

The aim of this systematic review and meta‐analysis was to analyse published RCTs that investigated the accuracy and advantages of ECG in PICC tip localization to provide evidence for clinical nursing use.

## 2 METHODS

This review was designed according to the framework recommended by the Cochrane Collaboration. We reported all results in accordance with the Preferred Reporting Items for Systematic Reviews and Meta‐Analyses statement (PRISMA; Moher et al., 2009). Research Ethics Committee approval was not required for this study because our study was conducted based on published data.

### 2.1 Eligibility criteria

This systematic review included all RCTs that investigated the use of ECGs to localize the peripherally inserted central catheter tip position. Eligible studies compared patients in whom the ECG localization technique was used with subsequent chest X‐ray to confirm the tip position. Studies were considered eligible for inclusion regardless of publication status, language or size.

Studies were excluded if they (a) were not RCTs, (b) did not compare ECG guidance technology and landmark positioning, (c) were in a language for which a translation to English was not available or (d) were unpublished studies with only the abstracts presented at national and international meetings.

The main outcomes of interest were the accuracy rate of catheter tip placement and the incidence of complications for the ECG and landmark methods.

To be eligible, in the intervention group, the location of the catheter tip was determined by ECG and chest X‐ray after catheterization; in the control group, the location of the catheter tip was determined by landmark and chest X‐ray after catheterization, and the determination index of the catheter tip position was as follows: (a) The tip of the catheter was in place, and the tip of the catheter was located in the middle and lower 1/3 of the superior vena cava. (b) The chest X‐ray film showed that the catheter tip was at the level of 6 (T6) – 8 (T8). (c) The ectopia of the catheter tip was identified when the tip was located outside the superior vena cava, such as in the subclavian vein, internal jugular vein and right atrium. (d) The tip of the catheter was too shallow such that the tip of the catheter was located in the middle and upper segments of the superior vena cava. (e) The landmark location means the patient is placed in a supine position, the upper limb on the tube side is kept on the same plane with the trunk, and the upper limb abduction is at a 90° angle with the trunk. A soft ruler is used to measure from the puncture point to the right sternoclavicular joint and then down to the third intercostal space. The sum of the measured values of the two segments is the PICC tube length. (f) ECG positioning technology is used to connect a special ECG lead wire to the catheter guide wire and the electrocardiograph and judge the position of the catheter tip by observing the characteristic changes of the P‐wave on the central electrogram during the catheterization process.

### 2.2 Search methods for the identification of studies

We searched for studies on MEDLINE, LILACS, EMBASE, SciELO, the Cumulative Index to Nursing and Allied Health (CINAHL), PEDro and the Cochrane Library up to July 2019, without language restrictions. A controlled vocabulary was used (MeSH terms for MEDLINE and Cochrane; EMTREE for EMBASE). Keywords and their synonyms were used to sensitize the search, including “peripherally inserted central catheter” or “PICC” or “PIC” and “electrocardiogram” or “electrocardiography” or “IC‐ECG” or “ECG” or “EKG” or “IC‐EKG”. The retrieval was conducted in the form of free words with a keyword.

For the identification of RCTs in PUBMED, the optimally sensitive strategy developed for the Cochrane Collaboration was used (Higgins & Green, 2006). For the identification of RCTs in EMBASE, a search strategy using similar terms was adopted. In the search strategy, there were four groups of keywords: study design, participants, interventions and outcome measures.

We analysed the reference lists of all eligible articles to identify other potentially eligible studies. For ongoing studies or when any data were to be confirmed or additional information was needed, the authors were contacted by e‐mail.

The previously described search strategy was used to obtain titles and abstracts of studies that were relevant for this review. Each identified abstract was independently evaluated by two authors. If at least one of the authors considered one reference eligible, the full text was obtained for complete assessment. Two reviewers independently assessed the full text of the selected articles to verify whether they met the criteria for inclusion or exclusion. In case of any disagreement, the authors discussed the reasons for their decisions, and a consensus was reached.

Two authors, independently blinded, extracted descriptive and outcome data from the included studies using a standardized form developed by the authors and adapted from the Cochrane Collaboration's (Higgins & Green, 2006) model for data extraction. We considered (a) aspects of the study population, such as the average age and sex; (b) aspects of the intervention performed (sample size, type of stabilization exercise performed, presence of supervision, frequency and duration of each session); (c) follow‐up (if the patients included were analysed); (d) loss to follow‐up (if there was a loss in the sample); (e) outcome measures; and (f) presented results. Another author resolved disagreements. Any additional information required from the original author was requested by e‐mail.

The quality of evidence was independently scored by two researchers based on the PEDro scale (Olivo et al., 2008), which consisted of 11 items based on the Delphi list. One item on the PEDro scale (eligibility criteria) is related to external validity and is generally not used to calculate the method score, leaving a score range of 0–10.

### 2.3 Statistical assessment

The meta‐analysis was performed with RevMan (Version 5.3. Copenhagen: The Nordic Cochrane Centre, The Cochrane Collaboration, 2014), and RRs and 95% CIs were used to summarize the outcomes. First, the heterogeneity of the research problem was assessed by the Cochran test. The significance level for the Cochran test was set at *α* = 0.1 as recommended by authors such as Sedgwick (2015). We used Cochrane Q to qualitatively describe the heterogeneity across eligible studies, and then, the *I* ^2^ statistic was used to quantitatively estimate the heterogeneity. A low degree of heterogeneity was indicated by *p* ≥ .1 and *I* ^2^ ≤ 50%, and a high degree of heterogeneity was indicated by *p* < .1 and *I* ^2^ > 50%. If the heterogeneity was high, we performed a further analysis of the heterogeneity sources. If there was no significant clinical heterogeneity, the random effects model was used for the meta‐analysis. In this study, we performed all statistical analyses based on the random‐effect model because heterogeneity cannot be omitted in reality.

### 2.4 GRADE framework to rate the certainty of the evidence

The GRADE framework, a grading system for the quality of evidence, was used to classify the quality of evidence for outcome indicators. All RCTs were included in this study. RCT was set as the highest level of evidence. There are five factors that can reduce the quality of evidence: research limitations, publication bias, research inaccuracy, research inconsistency and indirectness of research results.

## 3 RESULTS

### 3.1 Identification of literature

**FIGURE 1:**
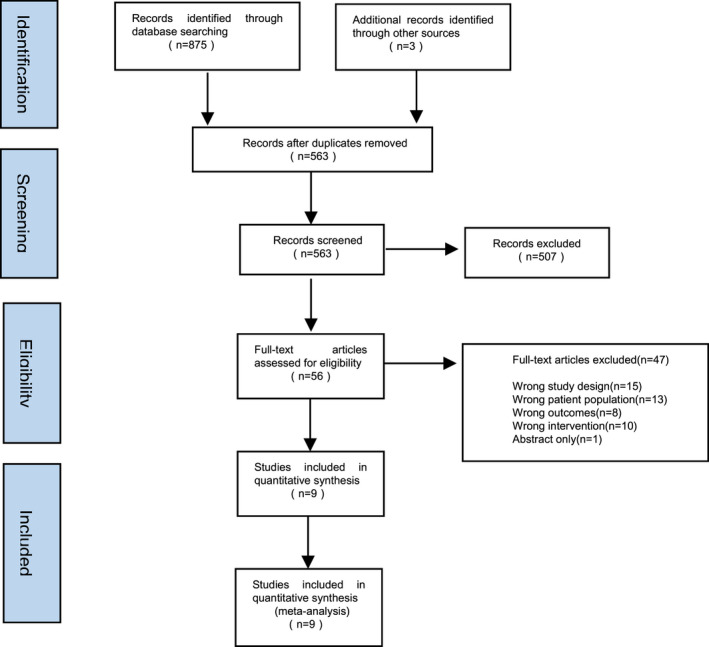
Search and selection of studies for systematic review according PRISMA

The initial search led to the identification of 875 abstracts, from which 56 studies were considered potentially relevant and retrieved for detailed analysis. After a complete reading of 56 articles, 47 were excluded. Finally, 9 papers met the eligibility criteria. Figure 1 shows the PRISMA flow diagram of study selection for this review.

### 3.2 Characteristics of all included studies

The characteristics of the included studies are documented in Table 1. All eligible studies were published between 2007–2017. Among these studies, 2 were conducted in Italy, 3 in China and 1 each in Sevierville, South Korea, India and the United States. The sample size of each individual study ranged from 60–1,003, and the mean age ranged from 34–41 years. All studies were published in academic journals in full text. Nine studies reported the ECG and landmark methods for localization of the catheter tip, and 7 reported the ECG and landmark methods for incidence of complications.

**TABLE 1:** Characteristics of the included studies

Study	Country	Participants ECG	Participants landmark	Type of trial	Type of Catheter	Criterion standard
Baldinelli et al., 2015	Italy	42	48	RCT	PICC	①②
Elli et al., 2016	Italy	75	44	RCT	PICC	②
Liu et al., 2015	China	85	85	RCT	PICC	①②
Zheng et al., 2015	China	513	515	RCT	PICC	①
Cales et al., 2016	Sevierville	102	85	RCT	PICC	①②
Lee et al., 2009	South Korea	121	128	RCT	PICC	① ②
Barnwal et al., 2016	India	30	30	RCT	PICC	①②
Gebhard et al., 2007	America	147	143	RCT	PICC	① ②
Yuan et al., 2017	china	499	504	RCT	PICC	①②

Note

①, ECG and landmark methods for localization of the catheter tip. ②, ECG and landmark methods for incidence of complications.

### 3.3 Risk of bias

Each of the studies was scored using the PEDro scale. Table 2 presents the results of the individual assessments by the PEDro scale. The overall quality of all studies was fair to good, but the therapists and participants were not blinded in the design of all articles. All articles followed eligibility criteria and source of participants; random allocation; baseline comparability; adequate follow‐up; between‐group comparisons; and point estimates and variability. Three articles lacked concealed allocation, 3 articles lacked blind assessors, and 2 articles lacked intention‐to‐treat analysis.

**TABLE 2:** Study quality on the PEDro scale

	Study	1	2	3	4	5	6	7	8	9	10	11	Total
1	Baldinelli et al., 2015	√	√	√	√			√	√	√	√	√	8
2	Elli et al., 2016	√	√	√	√			√	√	√	√	√	8
3	Liu et al., 2015	√	√	√	√				√	√	√	√	7
4	Zheng et al., 2015	√	√		√				√	√	√	√	6
5	Cales et al., 2016	√	√	√	√			√	√		√	√	7
6	Lee et al., 2009	√	√		√			√	√	√	√	√	7
7	Barnwal et al., 2016	√	√	√	√			√	√	√	√	√	8
8	Gebhard et al., 2007	√	√	√	√				√	√	√	√	7
9	Yuan et al., 2017	√	√		√			√	√		√	√	6

Note

1, eligibility criteria and source of participants; 2, random allocation; 3, concealed allocation; 4, baseline comparability; 5, blinded participants; 6, blinded therapists; 7, blind assessors; 8, adequate follow‐up; 9, intention‐to‐treat analysis; 10, between‐group comparisons; 11, point estimates and variability. Item 1 does not contribute to the total score.

### 3.4 Confirmed accuracy of the catheter tip position

**FIGURE 2:**
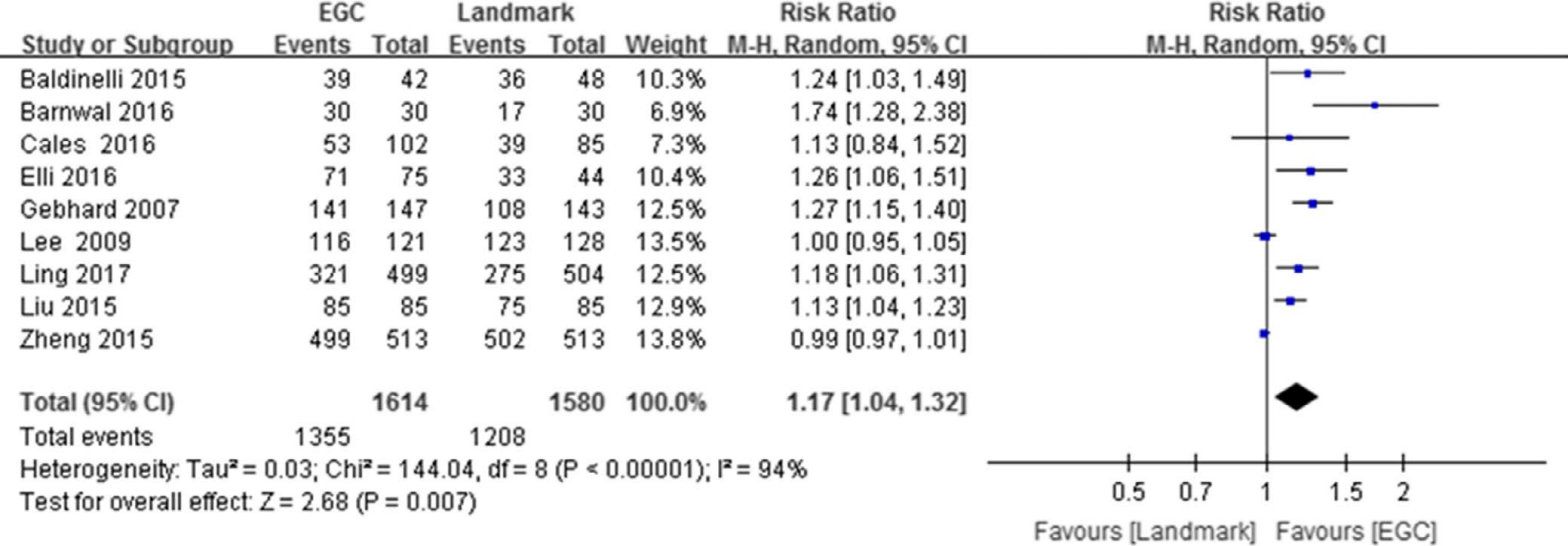
Forest diagram of ECG and landmark methods for localization of the catheter tip

In total, 9 trials assessed the accuracy of the catheter tip position. The meta‐analyses showed (Figure 2) a significant improvement in the accuracy of the catheter tip position (RR: 1.17, 95% CI: 1.04–1.32, *N* = 3,194) for participants in the ECG group compared to those in the landmark group.

### 3.5 Incidence of complications

**FIGURE 3:**
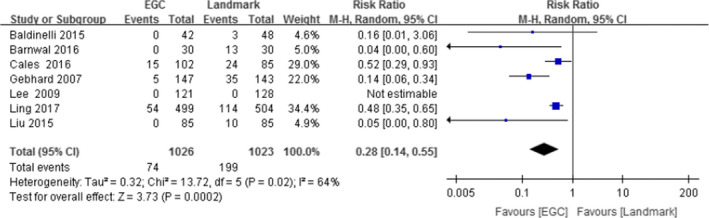
Forest diagram of ECG and landmark methods for incidence of complications

Seven trials assessed the incidence of complications. The meta‐analyses showed (Figure 3) a significant improvement in the incidence of complications (RR: 0.28, 95% CI: 0.14–0.55, *N* = 2049) for participants in the ECG group compared to the participants in the landmark group.

### 3.6 Sensitivity analysis

We reduced the included studies one by one and then analysed the remaining studies. The combined sensitivity and specificity results did not change significantly, indicating that the stability of the included literature was acceptable.

### 3.7 GRADE rating for outcomes

GRADEprofiler 3.6 software was used to grade the evidence for each outcome (Table 3). The evidence for all outcomes achieved medium quality.

**TABLE 3:** Summary table of outcome evidence

Study	Study design	Evidence quality evaluation					Summary of results				Importance
		①	②	③	④	⑤	Number of cases		RR(95% CI)	Quality of evidence	
							EGA	Landmark			
ECG and landmark methods for localization of the catheter tip											
9	RCT	serious	No	No	No	No	1,355	1,580	1.17(1.04–1.32)	B	important
ECG and landmark methods for incidence of complications.											
7	RCT	serious	No	No	No	No	1,026	1,023	0.28(0.14–0.55)	B	important

Note

① Iimitation; ② Inconsistency; ③ Indirectness; ④ Imprecision; ⑤ Bias of Publication.

B: Medium quality (A: high quality; B: Medium quality; C: low quality; D: Very low quality).

## 4 DISCUSSION

This systematic review and meta‐analysis included 9 eligible studies involving 3,194 patients to compare the ECG and landmark in localizing the peripherally inserted central catheter tip. Meta‐analysis of limited data suggested that the application of ECGs in PICC tip positioning can improve the accuracy of catheter tip positioning and reduce the incidence of related complications.

### 4.1 Analysis of the factors related to the production of the characteristic P‐wave

The characteristic P‐wave refers to the occurrence of a P‐wave amplitude change during PICC placement. When the catheter enters the superior vena cava, the amplitude of the P‐wave increases to 3.6 times that for the body surface. When the catheter reaches the right internal jugular vein (IJV), the P‐wave amplitude reaches its peak, which is 8.9 times that of the P‐wave amplitude for the body surface. When the catheter enters the middle and lower part of the right atrium, there is a negative P‐wave, and the negative P‐wave and Q‐wave can be W‐shaped (Pittiruti et al., 2008). In this study, while 58 patients (3.5%) did not produce a characteristic P‐wave, the PICC was confirmed to be in place by X‐ray for 33 of them. However, some studies have pointed out that approximately 0.7% of patients have no expected amplitude increase of the P‐wave (Pittiruti et al., 2012). Combined with the literature included, P‐wave formation is mainly related to the operator's manipulation, the guide wire of the conduit being too thin, poor conductivity of the catheter due to insufficient pre‐flushing of the catheter, incorrect injection of physiological saline when using the three‐way valve catheter, the opening of the three‐way valve at the end of the catheter to lead to the ECG of the cavity, and the difficulty in generating the characteristic P‐wave when the patient is positioned incorrectly.

### 4.2 The effect of electrocardiogram use on PICC tip localization accuracy

The results showed that there was a statistically significant difference in the accuracy of the catheter tip position between the ECG and landmark methods, which means that atrial ECG improves the accuracy of PICC tip localization, reducing the number of catheter adjustments after the destruction of the sterile environment; it can also reduce the rate of catheter tip ectopia. In the included studies, the rate of first‐attempt success by electrocardiography was 84.6% (*N* = 1,365), which is different from other scholars' reports (Hong & Lei, 2017; Oliver & Jones, 2016). Saager's study showed a correct placement rate of 95.5% if guided by ECG (Saager et al., 2007). The traditional chest X‐ray localization method is mainly based on the predicted length in vitro before the puncture and then chest X‐ray film inspection after the puncture. This method is easily affected by the measurement method of the technician, the change of the puncture point and the subjective factors of the technician; therefore, the catheter tip could be too deep, too shallow or ectopic. Once this situation occurs, the catheter tip cannot be recognized by the naked eye. Therefore, chest X‐ray film examination should be avoided for patients with characteristic P‐waves by ECG localization during catheterization with a PICC. In cases where a characteristic P‐wave is not observed, a chest X‐ray should be carried out to locate the catheter tip, and the catheter tip position should be determined and adjusted as required before use.

### 4.3 The application of atrial electrocardiogram for PICC tip localization reduced the incidence of related complications

The results from the included studies showed that the incidence of related complications was greatly reduced by using atrial ECG localization compared with the traditional localization methods.

The ECG localization method has the advantages of accurate and simple localization and is suitable for infants, pregnant women and elderly patients. Regarding the complications of ECG localization, some studies have noted that no complications or adverse reactions, such as arrhythmia, related to electrocardiographic technology were observed during catheterization. However, the few studies of the adverse reactions of ECG localization during and after catheterization must be verified by further clinical trials. The results show that in the process of catheterization with PICC, the tip of the catheter cannot be accurately placed in the ideal position due to some patients' vascular anatomy variation and disease characteristics. After the tip of the catheter is ectopic, it must be repeatedly adjusted, increasing the incidence of complications such as mechanical phlebitis, catheter‐related blood flow infection and thrombus (Capozzoli et al., 2012). For specific patients, such as patients with pleural effusion and ascites due to the upward movement of the heart position, when the catheter is delivered to the predicted length based only on in vitro measurements, the catheter head and catheter tip may already be located in the right atrium, which may lead to serious complications such as arrhythmia, heart thrombus, pericardial tamponade and pericardial rupture; once these occur, they can endanger the life of the patient (Rosche & Stehr, 2018; Walker et al., 2015).

### 4.4 Limitations of this study

There are few studies with limited sample sizes in this analysis. The resources used in this meta‐analysis research are from the literature, so it is impossible to obtain more detailed information. Because the literature is restricted by objective factors such as the source, the amount of information provided and the control of confounding factors, to a certain extent, it may have an impact on the conclusions of this study. Therefore, it is necessary to carry out prospective research focussing on the control of confounding factors while evaluating this question more scientifically and comprehensively. In addition, in clinical trials, there are many uncontrollable factors, so it is difficult to achieve randomization, assignment scheme concealment and blinding; therefore, the analysis results of this study are only for reference.

We must acknowledge some limitations in this systematic review and meta‐analysis. First, most of the eligible studies were underpowered due to inadequate sample size, which may reduce the robustness of the pooled results. Second, we did not set up the subgroup analysis in terms of time, mainly because the number of eligible studies was not sufficient. Third, some eligible studies did not describe the details of the ECG positioning technique, which will impair the value of clinical reference. Fourth, in the 9 RCTs included in this meta‐analysis, with sample sizes ranging from 60–1,028, there is heterogeneity in the methodology and sample. Fifth, the evaluation of methodological quality of all articles is not high, and the therapists and participants are not blinded in the design of all articles. Therefore, the quality of RCTs should be improved in the future.

## 5 CONCLUSION

The existing research shows that the application of atrial ECG in PICC tip positioning can improve the accuracy of catheter tip positioning, reduce the incidence of related complications and provide a scientific basis for further promotion of the atrial ECG positioning method. However, a definite conclusion on this topic cannot be drawn due to limited data. It is necessary to carry out multi‐centre, large sample follow‐up studies in the future to further explore and confirm the advantage of atrial ECG in PICC tip localization.

## CONFLICT OF INTEREST

The authors declare no conflicts of interest.

## AUTHOR CONTRIBUTION

Yu and Chunmei: Conceptualization and design. Yu and Juan: Data analysis. Ling and shulan: Writing the first draft. Both authors approved the final version of the manuscript.

## ETHICS APPROVAL

This study was approved by the ethics committee of Army Medical University.

## Data Availability

The data that support the findings of this study are available from the corresponding author upon reasonable request.
